# Annotations

**Published:** 1891-05-30

**Authors:** 


### Annotations.

**Another Lupus Cure.**

Chloride of gold is one of several new cures that have sprung into use as the result of Koch's new departure. The chloride is injected subcutaneously after the fashion of Koch's lymph. The fluid used is exceedingly attenuated, consisting of a one per cent. solution of chloride of gold in combination with a one per cent. solution of cyanide of potassium. One interesting case is reported; it is that of a peasant woman forty years of age, whose face, nose, forehead, cheeks and chin were studded with ulcers, the discharge from which contained tubercle bacilli. The treatment extended over eleven days, and six injections in all were given. The total quantity of the salts injected only amounted to $\frac{33}{1000}$ of a grain of each. The woman began to feel better on the second day of the treatment; and on the third the skin symptoms showed favourable modifications. On the sixth day some of the ulcers on the cheeks had begun to heal, and the pain and swelling of the face had disappeared. On the twelfth day several of the ulcers were quite healed, and the rest were in the process of recovery. Unfavourable symptoms resulting from the treatment were drowsiness, shivering, some elevation of temperature, and an appearance of intoxication. These symptoms lasted about twenty-four hours. It is well to note even a single case of this kind, although nothing can be founded thereupon. It seems evident that in order to cure lupus it is necessary to bring about a considerable exaltation of general physiological activity. It appears also that many substances subcutaneously injected are capable of bringing about such physiological activity. The line of research to be followed would seem to be the discovering of substances which will produce the greatest degree of exalted physiological activity, without the accompaniment of any injurious or dangerous circumstances.

**Sixty Thousand Suicides.**

Suicide is a cowardly performance, and a result of intellectual degeneracy. And yet, according to Dr. William Mathews, who writes in the *North American Review*, there are sixty thousand persons in Europe alone who are known to die of suicide every year, and probably at least sixty thousand more whose lives are terminated by suicide, though the facts are not definitely ascertained. Dr. Mathews thinks that over-civilisation and over-mental culture are responsible for this state of things. One, however, of the chief causes is the want of a religious faith which will firmly guide the life in a reasonable direction. Dr. Mathews gives a long list of historical names in support of his theory; and among others he mentions Aristotle, Cleanthes, Demosthenes, Zeno, Themistocles, Petronius Arbiter, Cato, Brutus, Cassius, Terence, Seneca, Aristarchus, Nero, Mithridates, Hannibal, Castlereagh, Kleist (the German author), Chatterton, Dr. Bull (author of England's National Anthem), Romilly, Hugh Miller, Colonel Gurwood, Haydon (the painter), Colton (author of "Lacon"), Prévost-Paradol, and H. B. Wallace. It is a striking fact that among all these illustrious persons there is not one whose religion was his most marked characteristic. It is certain that those who make a simple form of Christianity the authoritative guide of their lives practically never commit suicide. This is a fact which unprejudiced men of science cannot help considering. The business of science is to see facts, and to see them without prejudice, and in their true proportions and significance. Great historical facts, for example, are surely more important than facts that merely have to do with the wings of flies or the buccal arrangements of the coelenterates. The world in the main lives by large generalisations. An age that is deficient in generalisers is an age of intellectual poverty among the few, and of universal mental starvation among the masses. When populations are small and people live mainly on the soil and by the soil, great intellectual and scientific guides are comparatively little needed. But when the world consists almost entirely of rushing crowds, there is nothing so indispensable as powerful generalising minds, that can compel attention and lead the crowds in safe directions, so that they shall not trample one another to death in endless confusion. Christianity does two things for those who accept it practically, and apart from its metaphysics and its ecclesiasticisms: it makes them industrious, and it makes them contented. Now of all the sovereign remedies against suicide none are more sovereign than industry and contentment. Work alone is salvation—work with contentment is heaven. We are not arguing here for or against Christianity. But if a physician cannot afford to ignore any therapeutic resource which has proved itself invaluable to the multitude during hundreds of experimental years, still less can the practical scientist and statesman afford to despise ideas, whether religious or otherwise, which preserve human sanity, human happiness, and human life. Whether it actually be so or not, it seems to some of us who live in this age that the scepticism of the cultured is a mark of intellectual feebleness, a clear evidence of debilitated brains. And the mental weariness, despair, and suicide which result from over-culture and scepticism are to us convincing proofs that our contention is probably just. Men of sound and healthy brains do not commit suicide. Self-destruction is one of the strongest possible evidences of exhausted physical and mental energy. Is it not then the truth that a really worthy science, instead of grubbing for ever among wretched little details, will set itself to lay down broad and simple lines of life for the many? Can it be true in this age that the scientist is such a narrow-hearted, thin-blooded, vain, and unwholesome creature that "he hates," in well-known phrase, "the profane multitude"? Is this the last word of science? If it be, then of all the intellectual movements that the world has ever seen the scientific movement is the least worthy and the most fruitless. Sixty thousand suicides, or, to be nearer the truth, a hundred and twenty thousand suicides annually in Europe alone, constitute one of the most staggering facts that modern science has to deal with. Can it deal with this fact successfully? If it cannot, the days of modern science as a leading force are numbered; and scientific men, instead of taking the front rank, will have to walk very meekly, and far in the rear.

**Small-pox and Cow-pox.**

At the present time, when vaccination is on its trial, every fact bearing upon the subject is of importance. It should be remembered that whilst vaccination concerns the medical profession a little, and as a matter of daily business, it concerns the masses of our civilised populations a great deal, and as a question of life and death. One of the chief points in the present controversy is the identity or non-identity of small-pox and cow-pox. Is cow-pox a true, though modified, descendant of human small-pox? If it be, a strong argument is at once established in favour of vaccination. For the contention is that ordinary arm to arm vaccination is really, as the name imports, inoculation with the small-pox virus, after that virus has been passed through, and favourably modified by, the organism of the cow. M. Eternod and M. Haxiers have recently tried the experiment of inoculating calves with human small-pox virus. If the theory be true that cow-pox is in origin the same as human small-pox, and is simply modified by the cow, the inoculation of any bovine animal by human small-pox virus ought theoretically to produce cow-pox in the particular animal inoculated. What are the actual facts? These, that the first inoculation does not produce typical cow-pox; but that the transference of the lymph from the animal first inoculated to other animals in succession does at length result in an eruption which it is impossible to distinguish from true cow-pox. If this be indeed so, then one fact, as we have said, is established which is presumptive evidence in favour of the value of vaccination. But by a further experiment a still more important fact was demonstrated. The calves that were inoculated with the human small-pox virus, and also those other calves which were inoculated from the first set, and still other calves in continuous succession, proved themselves to be insusceptible to ordinary vaccine virus. That is, those animals that had been inoculated with the virus of the second, third, fourth, and other calves successively, as well as the originally inoculated animal, proved themselves to be insusceptible to vaccination with ordinary vaccine lymph. These experiments are clearly of very great value as bearing upon the present controversy; and it is much to be desired that they be repeated independently by a considerable number of competent persons in this country.

